# Understanding the Immune-Stroma Microenvironment in B Cell Malignancies for Effective Immunotherapy

**DOI:** 10.3389/fonc.2021.626818

**Published:** 2021-03-25

**Authors:** Benedetta Apollonio, Nikolaos Ioannou, Despoina Papazoglou, Alan G. Ramsay

**Affiliations:** Faculty of Life Sciences & Medicine, School of Cancer and Pharmaceutical Sciences, King’s College London, London, United Kingdom

**Keywords:** immunotherapy, tumor microenviroment, stroma, anti-PD1, lymphoma, interferon, T cells, CAR T

## Abstract

Cancers, including lymphomas, develop in complex tissue environments where malignant cells actively promote the creation of a pro-tumoral niche that suppresses effective anti-tumor effector T cell responses. Research is revealing that the tumor microenvironment (TME) differs between different types of lymphoma, covering inflamed environments, as exemplified by Hodgkin lymphoma, to non-inflamed TMEs as seen in chronic lymphocytic leukemia (CLL) or diffuse-large B-cell lymphoma (DLBCL). In this review we consider how T cells and interferon-driven inflammatory signaling contribute to the regulation of anti-tumor immune responses, as well as sensitivity to anti-PD-1 immune checkpoint blockade immunotherapy. We discuss tumor intrinsic and extrinsic mechanisms critical to anti-tumor immune responses, as well as sensitivity to immunotherapies, before adding an additional layer of complexity within the TME: the immunoregulatory role of non-hematopoietic stromal cells that co-evolve with tumors. Studying the intricate interactions between the immune-stroma lymphoma TME should help to design next-generation immunotherapies and combination treatment strategies to overcome complex TME-driven immune suppression.

## Introduction

There is a clinical need to identify novel treatments for lymphoid malignancies (1). Effective immunotherapy promotes the killing of cancer cells by cytotoxic T cells. Immune checkpoint blockade has demonstrated that reinvigorating anti-tumor immune activity can induce durable responses across multiple cancer types and serves as an illustrative example of therapeutically targeting the tumor microenvironment (TME) (2). The most promising clinical responses to PD-1 blockade have been seen in classical Hodgkin lymphoma (cHL) and primary mediastinal B cell lymphoma (PMBL) with up to 87% overall response rate (ORR) detected in relapsed/refractory (R/R) cHL (3). However, the efficacy of anti-PD-1 immunotherapy in non-Hodgkin lymphomas (NHLs) including diffuse large B-cell lymphoma (DLBCL) and follicular lymphoma (FL) has been more modest (4). Unexpectedly, no activity was seen in a trial of anti-PD-1 therapy for R/R chronic lymphocytic leukemia (CLL) although PD-L1-PD-1-mediated T cell dysfunction has been described (5–7). This clinical experience suggests that profound immunosuppressive barriers operate within the TME.

The current immuno-oncology era is directing attention towards the relevance of studying the composition and function of the immune TME, together with genomic analysis. In this review, we discuss the current understanding concerning the role of T cells and inflammatory signaling (“inflamed” *versus* “non-inflamed” immune TMEs) in generating both endogenous anti-tumor immune responses, as well as sensitivity to immunotherapy. We describe how cancer immunology has been the subject of intense research in the solid tumor field but has been comparatively ill-defined in the lymphomas. We highlight tumor intrinsic and extrinsic mechanisms of resistance in generating an effective anti-tumor immune response, before introducing an understudied area of lymphoma research—the role of non-hematopoietic stromal cell types in regulating anti-tumor immunity, that could also represent a targetable obstacle that immune cells face in the cancer-immunity cycle.

## The Immune TME and Anti-Tumor Immunity

It is known that the immune system can paradoxically both inhibit and promote tumor development through a dynamic process where complex interactions between malignant cells and the immune system determine the fate of tumors, termed cancer immunoediting, which progresses through three phases: elimination, equilibrium, and escape (8, 9). During the elimination phase, the immune system recognizes and eradicates transformed cells; however, some tumor clones can avoid elimination leading to the equilibrium phase during which tumor growth is kept in check (10). However, chronic inflammation as well as evolutionary pressure from immune cells, can allow tumor sub-clones to escape immune surveillance, leading to tumor outgrowth and disease (10). Although cancer immunoediting has been defined using murine models, next generation technology is now in a position to shed light on the relevance of these concepts for human patients and clinical observations (11).

The immune composition of the TME is a major determinant of tumor progression through these phases and includes innate (natural killer cells, macrophages, neutrophils, mast cells and dendritic cells), and adaptive (T and B cells) immune cells. The composition of the TME in B cell lymphomas that arise in secondary lymphoid tissues, can vary from resembling normal reactive lymph nodes with germinal centers as seen in FL, to tumor effacement as exemplified by CLL and DLBCL (12). In common with solid cancers, malignant cells actively influence the composition of the TME and educate surrounding immune and stromal cells that can acquire pro-tumorigenic and immunomodulatory activity.

Particular cell types of the innate and the adaptive immune system can function in a tumor-promoting or inhibitory way with neutrophils, M2-polarized tumor-associated macrophages (TAMs), T_H_2 CD4^+^ T cells and T_Regs_ generally considered as pro-tumor cells, whereas, M1-macrophages/TAMs, T_H_1 CD4^+^ T cells and cytotoxic CD8^+^ T cells are associated with anti-tumor functions (13). Importantly, in a significant proportion of cancer patients, including the lymphomas, there is evidence of an active anti-tumor immune response directed against tumor-specific (neoantigens) (14, 15) or tumor-associated antigens (16). However, although acute inflammatory signals can stimulate adaptive immunity, chronic inflammation can be antagonistic and promote immune suppression. Numerous studies in solid cancer have shown that an activated adaptive immune response involving effective antigen presentation and interferon (IFN) signaling, as well as sufficient numbers of T cells in the TME, is associated with a favorable prognosis (13, 17, 18).

### T Cells and Anti-Tumor Immunity

The crucial role of T cell lymphocytes as the main regulators and effectors in anti-tumor immunity has been well established. Seminal studies have revealed the role of immune surveillance and the vital contribution made by adaptive immune cells in suppressing the formation of tumors (19–21). Murine models have shown that tumor killing is mainly mediated by cytotoxic CD8^+^ T cells in both solid (13) and B cell lymphomas (22). Elegant studies using the transgenic Eµ-TCL1 mouse model of CLL have shown that CD8^+^ T cells play an important role in controlling disease development in an IFN*γ*-dependent manner (23). CD8^+^ T cells, following successful priming, recognize antigens presented by tumor cells on their surface in complexes with HLA class I molecules and kill their targets, primarily *via* the release of cytotoxic molecules such as perforin and granzymes (24, 25). While the anti-tumor role of CD8^+^ is generally accepted, the role of CD4^+^ T cells in cancer immunity is controversial. T_H_1 CD4^+^ T cells, *via* the secretion of pro-inflammatory cytokines such as IFN*γ* and IL-2, can activate antigen presentation and costimulatory function on antigen-presenting cells (APCs), promote the effector differentiation of CD8^+^ T cells and enhance their migration (26). However, as mentioned above, some subsets of CD4^+^ cells can exhibit pro-tumor functions. T_H_2-polarized CD4^+^ T cells secrete cytokines that can limit CTL differentiation and proliferation such as IL-10 and IL-4, while CD4^+^ T_Regs_ (FOXP3^+^) have significant immunosuppressive functions through various mechanisms and their pro-tumor role has been described in both solid tumors and lymphomas (13, 27–29). In CLL, *in vitro* and xenograft murine models have demonstrated a pro-tumor effect of the CD4^+^ T cell compartment in patients, with correlations of CD4^+^ subset counts and clinical outcome supporting this role (28, 30). However, research using Eµ-TCL1-based murine models has suggested a more complex role of CD4^+^ T cells with some studies showing an anti-tumor function (31), while others demonstrating a dispensable role (23). Such differences likely reflect differences in the assay systems used, and the degree to which they model complex immune TMEs, as well as highlighting the divergent role of T_H_1 *versus* T_H_2-polarized CD4^+^ T cell subsets and their plasticity. Interestingly, CD4^+^ T cells specific for immunoglobulin-derived neoantigens in mantle cell lymphoma (MCL) were shown to express granzyme B and possess cytolytic function against autologous tumor cells following their engagement and expansion (14, 15), as previously described for granzyme B^+^ tumor-reactive CD4^+^ T cells in solid cancer (32). However, despite their crucial roles in controlling tumor growth, T cells become dysfunctional due to exhaustion in the chronically inflamed TME. T cell exhaustion is characterized by low proliferative capacity and limited effector function, due to chronic tumor antigen exposure (33, 34). This, in combination with a multitude of additional mechanisms covered later in this review, allow some tumors to escape both endogenous and therapy-mediated anti-tumor responses.

### The Importance of Type I and II IFNs in the Immune TME

Numerous studies have established the important role of the type II IFN, IFNγ as a key player in host anti-tumor immunity through direct anti-tumor and indirect immunoregulatory actions (19, 35–39). IFNs can suppress tumors directly by triggering pro-apoptotic signaling or inhibiting their proliferation, while increasing MHC expression (40), antigen presentation (41) and promoting tumor immunogenicity, a critical process for effective tumor recognition and elimination by the immune system (19). IFN*γ* strongly promotes inflammatory responses and has been shown to augment the function of tumor-infiltrating immune cells including CD4^+^ T_H_1 cells, CD8^+^ T cells, dendritic cells and macrophages, while suppressing T_H_2 cells and T_regs_ (20, 39). Despite its pleiotropic effects, it has been demonstrated that IFN*γ*-expressing T cells play a dominant role in mediating effective anti-tumor activity (42).

Although the majority of studies have used solid tumor models, the importance of IFN*γ* in anti-tumor immunity has also been shown in B cell malignancy models. Effective lymphoma immune surveillance using murine models was shown to be mediated by tumor-specific CD4^+^ T cells and associated with proinflammatory cytokines, particularly those that promote a T_H_1 phenotype including IFN*γ*, IL-2, and IL-12 (43). As discussed above, a non-redundant role of IFN*γ*-expressing CD8^+^ T cells in suppressing CLL progression was demonstrated using a murine transgenic model (23). Recent work has revealed that a subset of DLBCL harboring a T cell-inflamed TME (discussed later in this review) expressed a number of inflammatory and effector cytokine pathways including IFNγ and TNFα (44). Moreover, a T_H_1 cytokine profile including type II IFN T cell responses has been associated with a favorable prognosis and response to traditional chemotherapy, the immunomodulatory drug lenalidomide and anti-PD-1 immunotherapy in NHL patients (44–48).

Type I interferons (including IFN*α* and IFN*β*) can be produced by all nucleated cells and act on both tumor and immune cells (38, 49). Even though some decades-old studies had suggested an anti-tumor activity for IFN*α*/*β*, only recently, their role in the elimination of tumor cells and immunoediting has started to be elucidated (50, 51). Several studies have established their direct anti-tumor effect which is mediated through growth inhibition and/or induction of apoptosis by the regulation of the cell cycle (G1 phase arrest) and activation of both intrinsic and extrinsic apoptotic pathways (TRAIL, FAS and FASL among others) (52, 53). Moreover, type I IFNs have been shown to increase both HLA class I and tumor antigen expression and consequently, increase immune recognition and successful generation of an anti-tumor response (54, 55).

In addition to direct effects on tumor cells, there is substantial evidence showing that type I IFNs mainly function through stimulating anti-tumor immune responses that is relevant for both natural and therapy-induced immunity. The autocrine and paracrine circuits within the immune TME that are triggered by type I IFN show similarity with IFN*γ* signaling but they do not overlap completely (50, 56). Type I IFN deficient dendritic cells (DCs) in murine TME models have been correlated with ineffective T cell priming (57, 58). In common with IFN*γ*, the ability of type I IFN to promote immune-mediated anti-tumor activity is also through their effects on T cells. More specifically, type I IFNs have been reported to play an important role in T_H_1 CD4^+^ T cell polarization as well as promoting the survival and effector function of CD8^+^ cytotoxic T cells by increasing granzyme expression (51, 59, 60). In addition, type I IFNs have been shown to negatively regulate the numbers and activity of T_Regs_ (61) and myeloid-derived suppressor cells (MDSCs) (62).

Remarkably, in the context of hematological malignancies, one of the earliest studies indicating a role of type I IFNs in promoting immune-mediated anti-tumor responses was reported in a mouse lymphocytic leukemia model (63). Recent work has demonstrated that avadomide, a cereblon E3 ligase modulator (CELMoD), can induce IFN signaling in DLBCL B cells that triggered tumor apoptosis (64). In addition, the immunomodulatory drug lenalidomide has been shown to induce IFN*β* signaling in DLBCL that promotes tumor cell death (65). In contrast, lenalidomide and avadomide have anti-proliferative effects on CLL cells that are likely a direct effect of IFN signaling induction but does not induce direct tumor B cell apoptosis (66, 67). Interestingly, the fusion of IFN*α* to anti-CD20 antibody induced a superior anti-lymphoma effect than anti-CD20 alone by direct and potent killing of type I IFNα receptor-positive lymphoma cells (68). It was further demonstrated that an important additional mechanism of action of tumor-directed antibodies, in addition to direct anti-tumor effects, was the induction of type I IFN in the TME that activated DC cross-presentation and T cell activation (69). This work highlights the potential of next-generation Ab-based immunotherapy (Ab–IFN fusion) (70) to induce direct anti-tumor effects and reconnect suppressed innate and adaptive immune responses in the TME (69). However, it is worth noting that earlier work in CLL has described conflicting data regarding a pro-survival effect when treating CLL cells with recombinant type I (71–73) or type II IFNs (74), that may be associated with studies using peripheral blood-derived CLL cells, rather than tissue culture or *in vivo* models that mimic activated CLL TME biology (75). Recently, impaired IFN signaling in CLL B cells (loss of IFN regulatory factor 4, IRF4 using a murine model or its reduced expression in human CLL cells) has been linked to downregulated antigen presentation and co-stimulatory molecules that prevented the generation of activated, exhausted T cell responses and was associated with accelerated disease progression. In addition to this tumor immune evasion mechanism, T cells from treatment naïve CLL patients have been shown to express deregulated IFN type I and II signaling genes compared to healthy age-matched control T cells (67), in keeping with impaired IFN signaling in T cells representing a common immune defect in cancer (76). Together these recent findings provide evidence that reduced IFN signaling in the CLL TME could contribute the development of an immunosuppressive/non-inflamed TME.

### Inflamed *Versus* Non-Inflamed Immune TMEs

Analysis of solid cancer tumors including colorectal, melanoma, and ovarian cancer among others has established the prognostic significance of the type, density, and localization (immune contexture) of immune cells that reside within the TME for predicting a patient’s overall survival (77–80). The prognostic power of these variables is so powerful that they led to the development of a new scoring system termed the ‘immunoscore’, which has been internationally validated for colorectal carcinomas (CRC) (81). The immunoscore is based on the quantification and localization of CD3^+^ and CD8^+^ lymphocytes within tumors and their invasive margin and evidence so far suggests that this alternative scoring system is a more robust classification system for CRC for predicting survival than the classical tumor-node-metastasis staging system (81, 82). This was the first attempt of cancer classification/stratification through a system which was not tumor-based but rather immune-based, leading to a basic distinction of TMEs as T cell inflamed (or hot) that contain high numbers of infiltrated T cells, *versus* non-inflamed (or cold) phenotypes that are non-infiltrated (3, 18).

Transcriptome profiling studies have revealed additional molecular characteristics of T cell inflamed TMEs including the number of tumor-infiltrating T cells and the expression of IFN-inducible activated T cell biology gene signatures including granzymes, chemokines, PD-L1, as well as tumor mutational burden and neoantigen load (83, 84). In contrast, non-inflamed TMEs are generally characterized by low or absent infiltration of CD8^+^ cytotoxic T cells, low frequency of neoantigen expression, and a paucity of IFN signaling (18, 85). It should be noted that solid cancer and B cell lymphoma subtype TMEs fall into a spectrum of T cell inflamed to non-inflamed phenotypes reflecting expected heterogeneity, and can include “excluded” phenotypes where understudied stromal cells may present a barrier to infiltrating T cells (18). Applying the concept of inflamed *versus* non-inflamed TME phenotypes to lymphoma is challenging due to two major characteristics of these cancers. First, lymphomas are cancers of immune cells and as a consequence, tumor cells can regulate immunological functions themselves, making the tumor-immune cell interaction significantly more complex than solid tumors. Secondly, the origin and residence of malignant cells in most B cell lymphomas are secondary lymphoid organs that serve as sites of immune cell surveillance. Therefore, the presence of T cell subsets or inflammatory signatures in the TME may also represent active non-tumor immune responses. For example, cytomegalovirus causes a marked expansion of virus-specific T cells in CLL patients (86). However, the role of Epstein–Barr virus (EBV) in the pathogenesis of some lymphomas may also be a source of antigens for tumor T cell recognition (87). Regardless, a classification of lymphomas based on inflamed versus non-inflamed immune landscapes (‘immune contexture’) and responsiveness to immune checkpoint blockade has been described (3).

In the current era of immunotherapy, the characterization of tumors according to their immune landscape is more relevant than ever (88), as IFNγ-expressing T cell inflamed tumors have shown increased sensitivity to anti-PD-1 therapy with both predictive and prognostic significance (83, 89–91). PD-1 has been shown to be a negative regulator of pre-existing immune responses in tumors, thus blocking the interaction of PD-1 with its ligands (PD-L1 or PD-L2) prevents this inhibitory signaling and allows tumor-specific T cells to remain activated and kill tumor cells. Pre-clinical studies in solid cancer have suggested that anti-PD-1 therapy cannot function in the absence of primed tumor antigen-specific CD8^+^ cytotoxic T cells which express high levels of PD-1 (exhausted phenotype) (2, 92, 93). However, high-dimensional correlative analysis of circulating immune cells from melanoma patients receiving anti-PD-1 immunotherapy has revealed a more complex role of the T cell compartment during therapy. In particular, higher numbers of PD-1^+^, CTLA-4^+^, IL-17A^+^, IFN*γ* ^+^ activated memory CD4^+^ and CD8^+^ T cells including granzyme B^+^ CD4^+^ T cells were detected after therapy and in responding patients (94). Interestingly, the frequency of classical CD14^+^ HLA-DR^+^ monocytes prior to therapy was found to be a strong predictor of response, thought to reflect myeloid expansion triggered by IFN*γ* produced by activated tumor-specific and infiltrated T cells in patients who are more likely to become responders.

Inflamed lymphomas such as cHL tumors have shown increased sensitivity to anti-PD-1 blockade therapy and are characterized by an unusually high immune cell infiltrate, an inflammatory PD-L1^+^ TME, a high mutational burden (95) and genetic alterations that facilitate cancer immunoediting escape including aberrant MHC class I molecule expression (96). Intriguingly, the majority of T cells that infiltrate HL tumors are IFN*γ*-expressing T_H_1-polarized CD4^+^ T cells (96, 97), which is again at odds with the concept that PD-1 inhibition works solely through the reactivation of MHC class I-restricted CD8^+^ T cells (98), given that cHL is sensitive to this immunotherapy. Indeed, recent immune monitoring studies have shown that circulating cytotoxic granzyme B^+^, PD-1^+^ CD4^+^ T cells, as well as PD-1^+^ differentiated effector CD8^+^ T cells are detected in HL patients. Interestingly, only CD4^+^ T cell receptor (TCR) diversity at baseline and during therapy correlated with responses to anti-PD-1 therapy (99). Additionally, the study identified an IFN-experienced circulating CD68^+^ CD4^+^ Granzyme B^+^ innate effector subset in patients who responded to therapy. This correlative data highlights the potential cytotoxic anti-tumor capability of specific CD4^+^ T cell subsets that is relevant for both solid cancers (94) and lymphomas (99–102). It is also interesting to note that the presence of a baseline T cell inflamed TME has correlated with improved responses to other therapies including adoptive cell therapy in solid cancer models (103), that may have relevance for CAR T therapy in lymphoma (104), and the CELMoD avadomide which induced higher responses in immune-rich DLBCL TMEs (105). However, although a proportion of DLBCL (44) and FL tumors (106, 107) exhibit features of T cell inflamed TMEs, the immune landscape of the NHLs is more heterogenous with most harboring a cold or non-inflamed environment, and are typically resistant to anti-PD-1 therapy. A recent phase 2 study of anti-PD-1 in R/R DLBCL following autologous transplant reported an ORR of 10%, although some patients did show encouraging disease stabilization and durable responses (108). Non-inflamed lymphomas are thought to contain fewer infiltrating immune cells, particularly T cells, that can predict poor survival in DLBCL (109). Sparse infiltration of immune cells is linked to lymphoma intrinsic oncogenic pathways that prevent the recruitment or retention of immune cells in TME lymphoid tissues or downregulate APC capability (110). Interestingly, non-inflamed lymphomas typically lack genetic aberrations that facilitate immune escape (111). We have recently demonstrated that CLL lymph nodes show salient features of non-inflamed tumors including sparse numbers of CD8^+^ T cells relative to reactive non-malignant tissues and low PD-L1 expression in the TME, as well as profound T cell functional exhaustion (67).

## Tumor Intrinsic and Extrinsic Mechanisms That Influence Response To Immunotherapy

It is now clear that distinct mechanisms may confer sensitivity and resistance to different types of immunotherapy in tumors that show a large degree of heterogeneity in their immune TMEs, falling within the extremes of the inflamed *versus* non-inflamed classification. The key factors that shape the TME include tumor immunogenicity, oncogenic pathways, and genetic alterations that regulate T cell infiltration and function (3, 112). The sculpting of the immune TME is inherently interconnected with the cancer immunoediting process. Available evidence from studies of patients treated with immune checkpoint blockade drugs suggests that immunoediting takes place not only during tumor progression but, at least in some form, also in response to therapy (9). Several factors of immune escape and resistance to immunotherapy (innate or acquired) that have been characterized to date, can be broadly divided in tumor-intrinsic and tumor-extrinsic mechanisms (113, 114). While the bulk of evidence regarding the role of these processes in regulating anti-tumor immunity and response to immunotherapy comes from solid tumor research, recent studies are revealing similar mechanisms operative in the lymphomas.

### Tumor Cell-Intrinsic Mechanisms That Shape the Immune TME

**Figure 1:**
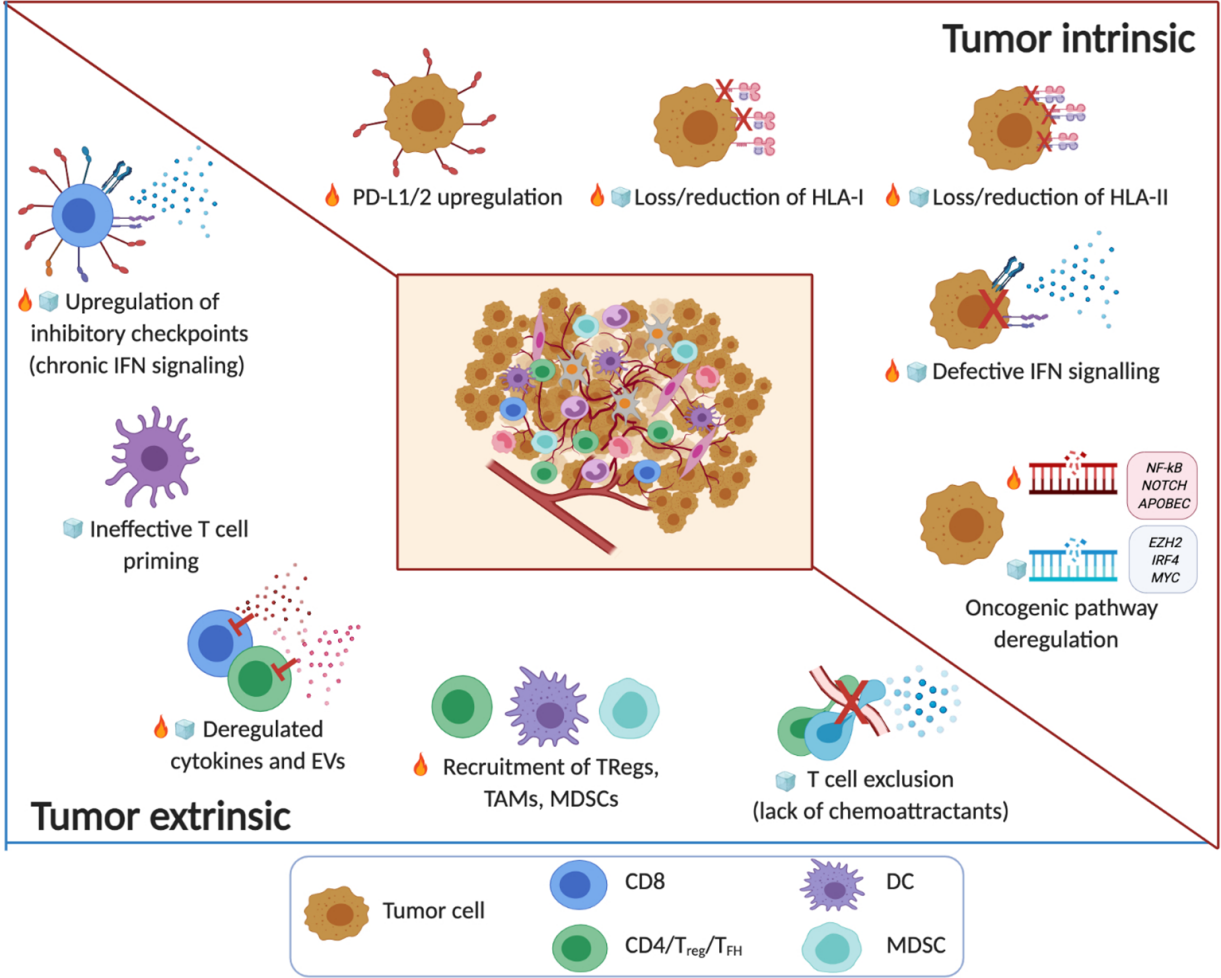
The immune TME in B cell malignancies. Tumor intrinsic and tumor extrinsic mechanisms associated with noninflamed/cold (
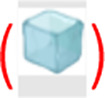
) or inflamed/hot (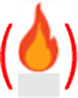) TMEs in B cell malignancy. The key factors that shape the TME include tumor immunogenicity, oncogenic pathways and genetic alterations that regulate T cell infiltration and function. Note: the timeline or sequence of events during the evolution of the altered pro-tumor, yet immunoprivileged TME is a current topic in the field. Emerging evidence from patients treated with immune checkpoint blockade drugs suggests that cancer immunoediting takes place not only during tumor progression but also in response to therapy (*e.g.* the acquisition of mutations that contribute to ‘defective IFN signaling’) (8–11). Several factors of immune escape and resistance to immunotherapy (innate or acquired) that have been characterized to date, can be broadly divided in tumor-intrinsic and tumor-extrinsic mechanisms. Tumor intrinsic mechanisms generally include genetic aberrations that can affect antigen recognition (‘loss/reduction of HLA-I/-II molecules’) (16, 115–119) and influence immune function (‘PD-L1/2 upregulation’) (120–122) and immune contexture in TMEs (‘oncogenic pathway deregulation’) including neoantigen load (83, 84). Tumor cell extrinsic factors that regulate anti-tumor immunity, immune evasion or resistance to immunotherapy involve non-tumor cellular and molecular components within the immune TME including ‘upregulation of inhibitory immune checkpoints’ (linked to chronic IFN signaling) (123–125), the ‘recruitment of TAMs, MDSCs, T_Regs_’ and stromal cells, as well as ‘deregulated cytokines and EVs’ (126–130), ‘ineffective T cell priming’ and ‘T cell exclusion’ (131–135). (*Created with Biorender.com*).

Tumor intrinsic mechanisms generally include genetic aberrations that can affect antigen recognition and influence immune function and immune contexture in TMEs including neoantigen load (**Figure 1**). HLA class I and II are required for the display and presentation of tumor-associated antigens and consequently an effective adaptive anti-tumor response. Several studies have reported HLA class I and II molecule loss or downregulation in lymphomas through genetic, epigenetic or transcriptional mechanisms. Intriguingly, acquisition of recurrent genetic alterations in genes encoding antigen presentation machinery appears to be a shared characteristic of T cell inflamed lymphomas such as cHL (115) and a subset of DLBCLs (44). This likely represents a critical cancer immunoediting escape mechanism used by lymphoma cells to evade the effector activity of lymphoma-specific CD8^+^ and CD4^+^ T cells. Impaired or loss of expression of HLA class I molecules is frequently detected in HL and DLBCL but has not been reported or is extremely rare in indolent lymphomas such as MCL, marginal zone lymphoma and CLL (16, 115–118). The most common mechanism leading to this altered HLA expression is caused by mutations and deletions in the *β*2-microglobulin gene, although direct genetic alterations of the HLA I genes have also been reported (115, 117, 119). HLA class II molecule downregulation is also observed in HL and DLBCL and mediated mainly at a transcriptional level through inactivating mutations CIITA, as well as homozygous deletions of chromosome 6p21.3 (116, 136–139).

Another mechanism contributing to immune evasion in lymphoma is the genetic overexpression of PD-L1, that is also a common feature of checkpoint blockade-sensitive T cell inflamed TMEs including cHL and PMBL (3). Upregulated expression of PD-1 ligands has been found to be driven by genetic alterations driving amplification of structural variations (SVs) of the chromosome region 9p24.1 which contains the loci for *PD-L1*, *PD-L2* and *JAK2* (termed the PDJ amplicon) (120–122). PD-L1 expression is less prevalent in DLBCL, but *PD-L1* gene alterations have also been detected in a subset of DLBCLs, particularly the non-germinal center B cell (GCB) subtypes, harboring T cell inflamed phenotypes with high numbers of infiltrating T cells, downregulated HLA expression and upregulation of inflammatory NF-kB, TNFα and IFN*γ* gene pathways (44, 120, 140). Recurrent SVs in the 3′ UTR of the *PD-L1* gene, which are thought to stabilize PD-L1 transcripts leading to increased protein expression, have also been uniquely described in DLBCL (141).

As mentioned earlier, type I and II IFNs are important mediators of both innate and adaptive anti-tumor immune function and are key players in cancer immunoediting. Importantly, defective IFN signaling in tumor cells has emerged as a major tumor-intrinsic resistance mechanism during immune checkpoint blockade therapy (114, 142, 143). Loss-of-function mutations in IFN*γ* receptor signaling pathway genes *JAK1* and *JAK2* have been seen in melanoma patients who developed late relapses after initial successful anti-PD-1 therapy (144), as well as patients with primary resistance to this immunotherapy (145). Mutations in IFN*γ*-related genes were also observed in non-responsive patients following anti-CTLA-4 checkpoint therapy (146). This loss of IFN*γ* receptor signaling allows the tumor to evade the effects of IFN*γ* produced by anti-tumor T cells that would reduce tumor antigen presentation capability, decrease the expression of IFN-associated chemoattractants and promote insensitivity to the anti-proliferative and pro-apoptotic effects of IFN*γ* in tumor cells. Several studies have found evidence of deregulation of tumor-intrinsic IFN signaling in lymphoma but this has not yet been directly linked to immunotherapy resistance. Suppression of type I interferon by STAT3 has been described in non-GCB DLBCL, with inhibition of STAT3 activity using ruxolitinib inducing a synergistic growth inhibition effect when combined with the IFN-inducing immunomodulatory drug lenalidomide (147). Evidence for a CLL cell-intrinsic IFN signaling defect was described earlier in this review with low expression of IRF4 associated with inferior prognosis and associated studies supporting a novel tumor immune evasion mechanism (148). This IFN signaling defect reduced expression of tumor genes required to activate T cells including antigen processing and presentation, that could contribute to resistance to immunotherapies in this disease.

In addition to altering intrinsic tumor cell properties, oncogenic signaling has been found to contribute to the immune contexture of inflamed and non-inflamed TMEs. Genetic aberrations affecting MYC, p53 and NF-kB among other genetic events can dictate the immune landscape (84, 149). Importantly, pathways including WNT-*β*-catenin and MAPK signaling, as well as those associated with loss of PTEN, have all been implicated in driving intrinsic resistance to immune checkpoint blockade in solid tumors (114). Recent genomic studies in DLBCL have revealed associations between alterations in oncogenes or tumor suppressors including *PTEN*, *EZH2* and *TP53* and reduced expression of genes linked to immune cell activation (150). However, mechanistic data on how oncogenic alterations promote a non-inflamed immune TME or immunotherapy resistance in lymphomas is currently lacking. Double hit GCB DLBCLs with *MYC*, *BCL2*, and/or *BCL6* gene rearrangements have been shown to contain low number of infiltrating T cells (111). These lymphomas are enriched for *EZH2* activating mutations that have recently been shown to underlie acquired deficiency in MHC I and II expression and low T cell infiltrates in murine lymphoid TMEs (110). Interestingly, *MYC* has been shown to regulate the anti-tumor immune response in murine models of T cell acute lymphoblastic leukemia (ALL) and liver cancer by inducing the expression of CD47 and/or PD-L1 immune checkpoint molecules and recruiting TAMs, while excluding T cells among other mechanisms (151–153). However, it should be noted that the ability of oncogenes like *MYC* to control the recruitment and function of immune cells in tumors can be counteracted by other genetic aberrations as has been seen using the *Eµ-MYC* lymphoma model (149, 154), that highlights the complexity of defining the roles of oncogenic alterations in modulating immune cell landscapes both within and between complex molecular lymphoma subtypes. Conversely, oncogenes and tumor suppression genes can equally foster an inflammatory immune TME. One notable example in solid cancer is NF-kB that controls cell survival and proliferation, but also production of inflammatory cytokines (149). In common, recurrent genetic modifications that lead to NF-kB activation have been described in inflamed lymphomas including cHL, PMBL and a subset of T cell-rich DLBCLs that contain *PD-L1* SVs and downregulated HLA expression (44, 155, 156). In addition, oncogenic NOTCH signaling has been implicated in the regulation of inflammatory DLBCLs that harbor genetic immune escape mechanisms including inactivating *CD70* and *FAS* mutations (157–159). Overall, further studies involving pre-clinical murine models will be required to define the how specific oncogenic signaling pathways contribute to tumor-immune cell interactions and T cell recruitment in lymphoma TMEs.

Several studies have shown that tumor neoantigens can function as targets for anti-tumor T cells and there is a positive correlation between tumor mutational burden and response to immune checkpoint blockade across cancers (160–162). In particular, deficiency in DNA repair mechanisms has been found to be the main driver of genomic instability and has been associated with response to immunotherapy (163). Interestingly, mutations that lead to DNA mismatch repair defects and microsatellite instability, as well as APOBEC mutational signatures have been identified in PD-1 blockade sensitive ‘‘inflamed’’ lymphomas such as cHL and PMBL; however, these seem more rare in other B cell malignancies (164–166). On the other hand, low tumor immunogenicity with low frequency of neoantigen generation as is seen in CLL, is linked to reduced intrinsic sensitivity to immune checkpoint blockade (5, 167). However, recent antigen-presentation profiling work is revealing that B cell lymphomas including CLL can present immunoglobulin neoantigens (15), with preferential MHC-II presentation that has implications for promoting the cytolytic T_H_1 differentiation of neoantigen-specific CD4^+^ T cells with immunotherapy, as has been demonstrated in solid cancer (168).

Recent studies have demonstrated that metabolically active tumor cells chronically deprive the TME of essential nutrients that affect T cell effector function and promote the creation of a tolerogenic TME [metabolic reprogramming in the lymphomas has been reviewed elsewhere (169–171)].

### Tumor Cell Extrinsic Mechanisms That Shape the Immune TME

Tumor cell extrinsic factors that regulate anti-tumor immunity, immune evasion or resistance to immunotherapy involve non-tumor cellular and molecular components within the immune TME including inhibitory immune checkpoints, TAMs, MDSCs, T_Regs_ and stromal cells (**Figure 1**). Although tumor extrinsic components have been linked to cold or non-inflamed TMEs and response to therapy, it should be noted that their roles in cancer immunology are highly dynamic and context dependent, including the nature and duration of the driving forces implicated. For example, cytokines within immune TMEs including IL-10 (131), IL-6 (132) and TGFβ (133) or extracellular vesicles (EVs) (134, 135) are known to have complex, context-dependent effects on both immune and cancer cells. Although there is evidence that these secreted factors have relevant immunomodulatory activity in B cell malignancies including CLL (172–176) and FL (177), our understanding of the hierarchy and cooperation required between cytokines and chemokines or EVs and their cellular sources for the licensing of immune evasion or the promotion of anti-tumor immune responses is currently ill-defined.

Type I and II IFNs can act as double-edged swords in cancer, promoting both feedforward immune activation responses described earlier, as well as feedback inhibitory mechanisms. Importantly, persistent IFN signaling in cancer, in common with chronic virus infection, can be immunosuppressive by inducing PD-L1, IDO and LAG-3 in the immune TME (123–125). Indeed, prolonged type I and II IFN-driven expression of multiple immune checkpoint ligands, receptors and inhibitory pathways linked to exhaustion including PD-1, LAG-3 and TIM-3, have been shown to be upregulated on T cell subsets during adaptive or acquired resistance to immune checkpoint therapy (125, 178, 179). It is plausible that combination checkpoint inhibition may be able to bypass negative feedback and multiple inhibitory receptors as has been demonstrated using a CLL murine model with dual anti-PD-1 and LAG-3 blockade (180). Interestingly, CD8^+^ CAR T cells co-expressing PD-1 with either LAG-3 or TIM-3 were associated with poor responses in CLL, whereas patients who had complete and durable remissions were infused with CAR T cells containing lower frequencies of these exhausted phenotypes. Moreover, the clinical effectiveness of CAR T cells in CLL was increased when co-stimulatory receptor CD27^+^ was expressed on CAR T cells that may reflect a less exhausted and functionally competent T cell phenotype (‘intrinsic T cell fitness’) (181). This data supports the relevance of multiple inhibitory pathways induced by IFN signaling that could hinder CAR T therapy and promote therapy resistance.

TAMs are an important subset of terminally differentiated myeloid cells that regulate anti-tumor immunity and response to therapy in many cancers including lymphoma (126, 127). Early work demonstrated that TAMs promoted chemotherapy resistance in cHL and in DLBCL in the pre-rituximab treatment era (182, 183). However, TAMs have been shown to mediate antibody-dependent cellular phagocytosis of rituximab engaged malignant B cells, highlighting therapeutic context. Studies have demonstrated the capability of TAMs to directly suppress T cells through PD-L1 expression, as well as the production of cytokines such as IL-10 and TGF-*β* or enzymes that can limit effector activity (126). Interestingly, inflamed cHLs harbor high numbers of PD-L1^+^ TAMs, that presumably represent an active immune evasive strategy elicited within the TME to suppress lymphoma-specific PD-1^+^ CD4^+^ T cells (184). In addition, it has been shown that lymphoma cells can upregulate expression of the membrane anti-phagocytic protein CD47 that allows them to escape elimination by TAMs. Combining anti-CD47 blocking antibody with rituximab has elicited synergistic anti-tumor activity in pre-clinical models and early clinical results have been promising (185, 186). These studies highlight the potential to harness anti-lymphoma TAM activity that could be combined with immune checkpoint blockade therapy to re-educate their pro-tumor, immunosuppressive activity and optimize immunotherapy. In addition, MDSCs have also emerged as potentially important regulators of immune responses, through the production of several immunosuppressive factors (ARG1, NO, PGE2) (187), and their presence in solid cancer TMEs correlates with decreased efficacy of immunotherapies including checkpoint blockade and adoptive T cell therapy (128, 129). However, a role in contributing to immunotherapy resistance in B cell malignancy has not been defined to date (188). Notably, in CLL it has been demonstrated that MDSCs, characterized by a CD14^+^HLA-DR^lo^ phenotype, can be induced by malignant B cells *in vitro* to suppress T cell effector function and promote T_Reg_-differentiation mediated by upregulation of indoleamine 2,3-dioxygenase (IDO) (189).

T_Regs_ comprise a subtype of CD4^+^ cells which are mainly defined by the expression of FOXP3 and play an important role in maintaining self-tolerance (190). T_Regs_ suppress effector T cells by secretion of inhibitory cytokines including IL-10 and TGF*β*, as well as direct cell contact. Indeed, many murine studies of solid cancer have shown that the depletion of T_Regs_ cells from the TME can enhance or restore anti-tumor immunity (130). Studies in CLL using the TCL1 transgenic model indicated that partial depletion of T_Regs_ numbers did not impact on CLL disease progression but did result in enhanced CD8^+^ T cell functional capacity (191). Increased numbers of circulating T_regs_ have been found in CLL patients and correlated with disease progression (192, 193), and lymph node tissue was shown to harbor twice the number of these suppressive cells than the peripheral blood compartment (194). Most immune checkpoint molecules including PD-1 and CTLA-4 are expressed on T_Regs_ but the effects of checkpoint inhibitors on this T cell subset and treatment response remain unclear. Intriguingly, studies have suggested that anti-PD-1 immunotherapy might enhance the immunosuppressive function of T_Regs_ (130, 195), whereas anti-CTLA-4 inhibitors might deplete these cells (196). It is possible that T_Regs_ cells may coincide with lymphoma-specific T cells, indicating a potentially immune-responsive tumor. Correlative studies to determine the impact of T_Regs_ on clinical outcomes for lymphoma patients receiving immunotherapies should be informative, as well as the development of targeted treatment approaches to deplete these cells in order to activate anti-tumor immunity (130).

## Stroma Cells as A Key Tumor Cell Extrinsic Mechanism That Regulate Immune Responses in the TME

Emerging evidence has now demonstrated that the creation of a “cold” TME requires the coordinated intervention of several other non-immune cell types that co-evolve with the tumor. Cancer-reprogrammed stromal cells [endothelial cells (ECs) and fibroblasts] acquire altered features that promote tumor progression and contribute to immunosuppression (197). This is achieved by a plethora of tightly orchestrated mechanisms influencing both spatial organization and function of immune subsets within the TME.

### Evidence That Stromal Cells Regulate Anti-Tumor Immunity in Solid Tumors

Extensive studies of tumor-associated endothelial cells (TECs) in solid cancer are highlighting not only the significance of angiogenesis in disease progression but also the role of tumor-reprogrammed ECs in recruiting and polarizing immune cells in the TME (198). EC-mediated T cell trafficking, a crucial and highly dynamic process, is initiated by the release of chemokines, to attract circulating leukocytes, followed by selectin-dependent leukocyte rolling on the endothelium and integrin-induced trans-endothelial migration (199). Multiple steps in this tightly controlled process are disrupted in tumors.

The tumor vasculature has been found to be less functional with abnormal ‘leaky’ vessels (200), contributing to the hypoxic state that characterizes the majority of solid tumors. In this hypoxic environment, ECs release nitric oxide (NO) that suppresses the expression of leukocyte adhesion molecules (VCAM-1, ICAM-1) (201). Decreased ICAM-1 expression on TECs has been shown to impair T cell extravasation and has been associated with decreased numbers of tumor-infiltrating lymphocytes (TILs) (202).

Together with ECs, cancer-associated fibroblasts (CAFs), the resident fibroblasts activated in a chronic inflamed TME, have been shown to actively shape the immune infiltrate. The CAF secretome promotes recruitment and polarization of regulatory cells from both the innate and adaptive immune arms (203). Recently, a subpopulation of immunoregulatory CAFs (CAF-S1) has been functionally involved in the attraction, retention and activation of T_Regs_ in the breast cancer TME through the secretion of CXCL12 (204). Moreover, other CAF-derived factors like Chi3L1, MCP-1 and SDF-1 drive the recruitment of monocytes and their polarization into pro-tumoral, immunosuppressive M2-macrophages in breast and prostate cancers (205–207), while CAF-derived IL-6 has been shown to promote immunosuppressive neutrophils in hepatocellular carcinoma (208). In addition, CAF-driven abnormal extracellular matrix (ECM) deposition and remodeling have been shown to induce the physical trapping of anti-tumor T cells to prevent effective tumor access (209).

Besides their role in mapping the spatial organization of immune cells in the TME, stromal cells can also directly influence or suppress endogenous anti-tumor immune responses through additional mechanisms, including antigen presentation. ECs upregulate both MHC-I and MHC-II in response to inflammatory cytokines (such as INFγ) acting as semi-professional non-hematopoietic APCs (210). As a consequence, ECs can mediate Ag-specific stimulation of effector memory CD4^+^ and CD8^+^ T cells (211–215). Interestingly, Motz and colleagues found that FasL expression on the vasculature of human and mouse solid tumors induced specific killing of CD8^+^ TILs, but not T_regs_, thus skewing the lymphocyte infiltrate towards a more regulatory phenotype (216). Moreover, ECs can upregulate PD-L1 and PD-L2 molecules inhibiting T cell activation and cytotoxic capacity (217–220).

Along the same lines, CAFs also directly impact the cytolytic activity of anti-tumor effector T cells through a number of different mechanisms. Prostaglandin E2 (PGE2) and NO produced by tumor-associated fibroblasts dampen CD8^+^ T cells proliferation in pancreatic and breast cancer respectively (221, 222) and CAFs expressing tumor antigens promote CD8^+^ apoptosis *via* PD-L2 and FasL expression (223). Overall, these intricate studies have shown that stromal cells shape the TME by affecting the recruitment and function of different adaptive and innate immune cells in solid tumors, but accumulating evidence is now revealing that similar mechanisms are operative in B cell malignancies.

### The Importance of Stromal Cells in B Cell Malignancies

In lymphoid organs [lymph nodes (LNs), spleen and bone marrow (BM)] resident stromal cells engage in bidirectional interactions with lymphocytes that directly contribute to the shaping and function of the immune system (224, 225). Lymphocytes enter the LN *via* both afferent lymphatics formed by lymphatic endothelial cells (LECs) and peripheral circulation through high endothelial venues (HEVs). Lymphocyte homing to secondary lymphoid organs is coordinated by the expression of integrin and adhesion molecules, as well as chemokine–chemokine receptors on ECs lining HEVs (199). Emerging evidence also shows that LN ECs also acquire distinct immunomodulatory roles such as priming T cells (226), while BM ECs critically influence bone marrow remodeling and hematopoiesis, also contributing to the differentiation of immature B cells (225). Besides ECs, lymphocytes also closely interact with stromal cells of mesenchymal origin: mesenchymal stromal cells (MSCs) in the BM and both fibroblastic reticular cells (FRCs) and follicular dendritic cells (FDCs) in the LNs. MSCs primarily function to create a reticular network in the BM and to produce factors essential to lymphoid lineage development and differentiation. MSCs are also a key component of the regenerative system and they possess the ability to migrate to a damaged tissue, promote its repair and suppress the associated inflammation through a number of immunomodulatory factors (227). In the LN, FRCs and FDCs maintain immune system homeostasis by guiding the correct compartmentalization of immune cells through the display and secretion of chemokines and cytokines. They also actively participate in the control of immune responses by regulating the recruitment and the activation status of myeloid cells and lymphocytes using similar mechanisms to the ones described for MSCs (228). Thus, it is reasonable to hypothesize that stromal cells could similarly participate in shaping the immune TME, with potential contribution to the immunosuppressive state in B cell malignancies.

Several studies have shown that stromal cell architecture is altered in both the BM and LN tissue compartments of patients with different hematological malignancies (229, 230). Increased proliferation of VEGFR-1^+^ neovasculature has been observed in aggressive lymphomas (DLBCL and Burkitt’s lymphoma), and an elevated numbers of αSMA^+^ mesenchymal cells described in indolent NHLs including CLL and small lymphocytic lymphoma (SLL) (231). Despite this general distinction, the stromal landscape of hematological malignancies is far more complex, with evidence that both ECs and fibroblasts cooperate in shaping disease biology and promoting tumor progression. Angiogenesis contributes to CLL development and correlates with disease progression (232–234). In line with this observation, overexpression of vascular endothelial growth factor (VEGF) in tissue biopsies has been shown to be a promising prognostic factor for NHL (235). In aggressive DLBCL, Lenz and colleagues described two different stromal gene signatures (stromal 1 and stromal 2) associated with patient survival following CHOP and R-CHOP treatment. The ‘stromal-2’ signature comprised of angiogenesis-associated genes (including CD31, VEGF), increased tumor blood-vessel density and correlated with poor prognosis following R-CHOP. On the other hand, the prognostically favorable stromal-1 signature showed enrichment for fibroblasts and ECM-associated genes (236). The importance of fibroblasts in aggressive lymphoma was functionally demonstrated in the Eµ-Myc mouse model, where expanded FRCs created a pro-tumor niche directing the homing and survival of lymphoma cells in the spleen (237). It has also been demonstrated in CLL using the Em-TCL1 murine model, that splenic stromal cells co-evolve with disease, in particular an expansion and reprogramming of splenic fibroblasts that produce the pro-B cell cytokine CXCL13 (238). To complicate the picture further, endothelial and mesenchymal cells are intimately linked to each other as exemplified by PDGF-activated MSCs in CLL that were shown to up-regulate VEGF production and promote the ‘angiogenic switch’ associated with disease progression (239). The importance of crosstalk in CLL between tumor cells and stroma cells has been recently reviewed by Dubois et al. (240).

### Malignant B Cells Hijack Stromal Cells in the Lymphoma TME

**Figure 2:**
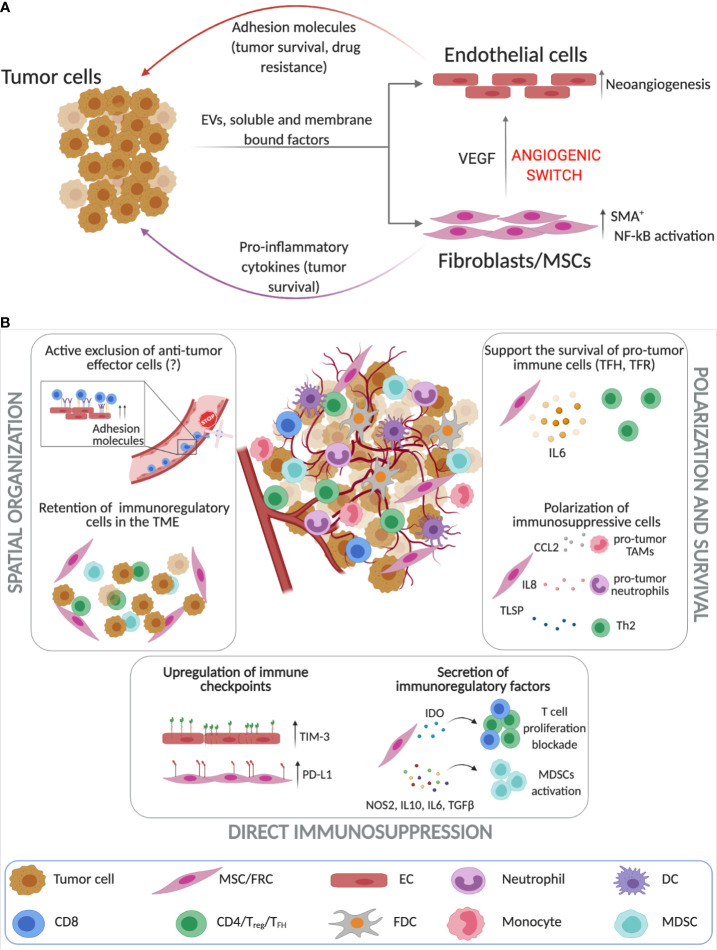
Stroma cells as key players in regulating immune responses in the TME of B cell malignancies. **(A)** Tumor cells and tissue-resident stroma (endothelial cells (ECs) and FRCs [fibroblastic reticular cells)/MSCs (mesenchymal stromal cells)] engage into complex bidirectional interactions that promote cancer progression while simultaneously altering the stroma cell phenotype which can then further contribute to resistance to therapy. Tumor B cells induce neoangiogensis and the upregulation of adhesion molecules on ECs (243–245, 248, 252). Similarly, lymphoma cells through cell-to-cell contact interactions, secretion of soluble factors and extracellular vesicles (EVs) promote the activation of FRCs and MSCs that contribute to increased tumor survival and neoangiogensis (135, 253–255). **(B)** Unlike solid tumors the investigation of the immunosuppressive roles of stroma cells in the lymphoma TME is still in its infancy. Stromal cells play a crucial role in spatial organization of the TME as they can retain immunoregulatory cells and possibly actively exclude anti-tumor effector cell populations (237, 253, 256–261). Additionally, lymphoma ECs and FRCs upregulate immune checkpoints such as TIM-3 and PD-L1 (261, 262) and secrete immunoregulatory factors such as IDO and IL-10 that block T cell proliferation, while activating immunosuppressive cells (263–266). FRCs have been also found to drive the survival of pro-tumoral immune subsets such as T_FH_, T_H2_ and TAMs (257–260). (*Created with Biorender.com)*.

Altered vascular patterns and angiogenesis characterize aggressive and indolent lymphomas (241, 242). Tumor B cells secrete pro-angiogenic factors such as VEGF (243, 244), that promote the activation of endothelial cells and neoangiogenesis. In turn, as highlighted by numerous studies in CLL (245–248) and other hematological malignancies (249, 250), activated ECs critically contribute to malignant B cell survival through factors such as B-cell activating factor (BAFF) (245, 251, 252) (**Figure 2A**).

Moreover, CLL and HL cells secrete EVs that promote the acquisition of a CAF-like phenotype in previously healthy ECs and fibroblasts from different origins (267, 268). Importantly, tumor-derived EVs can also modulate other cellular components of the TME (269), and their role in B-cell malignancies has been recently reviewed (135).

In multiple myeloma (MM), the combination of both soluble (SDF-1α) and membrane-bound factors (integrins) induce stroma activation (ECs and BM-MSCs) (263), while in FL and CLL, tumor-derived TNFα and lymphotoxin (LT) are involved in the remodeling of BM-MSCs and LN-FDCs respectively (253, 254). These tumor-specific factors ultimately converge in the activation of NF-kB-dependent transcriptional programs (253, 263) that promote the secretion of pro-inflammatory soluble mediators involved in the enhancement of cancer survival (255) and in the modulation of TME immune infiltration (**Figure 2A**).

### Evidence for the Immunomodulatory Roles of Reprogrammed Stromal Cells in B Cell Malignancies

As described above, tumor-activated stroma has a direct impact on the retention of tumor B cells and could therefore impact the recruitment or exclusion of other lymphocytes *via* similar mechanisms. The upregulation of adhesion molecules on EC surfaces (250) may negatively impact T cell trans-endothelial migration, which is a critical step of T cell homing into the LNs (270). In CLL, CD4^+^ and CD8^+^ T cells exhibit impaired integrin lymphocyte function-associated antigen-1 (LFA-1)–driven migration (271). Altered expression of adhesion molecules on TECs, together with T cell motility defects, could therefore block effective T cell trafficking into LN tissues.

Although studies showing a direct impact of activated endothelium on T cell recruitment in hematological malignancies are lacking, recently de Weerdt et al. revealed that CLL LNs contained twice the amount of T_Regs_ and a lower frequency of cytotoxic lymphocytes compared to the peripheral blood compartment (194). Under inflammatory conditions, activated ECs induce the expansion of T_Regs_ (215, 216) and the proliferation of memory CD4^+^ T cells (272), and it could be speculated that similar mechanisms occur in the CLL TME.

Despite the lack of evidence for ECs, several studies have demonstrated the contribution of mesenchymal cells in the regulation of immune infiltration in B cell malignancies. In a mouse model of aggressive lymphoma, where tumor cells rely less on the TME for survival, the co-injection of MSCs with tumor cells induced a marked increase of immune cells including CD4^+^ T cells, CD11b^+^ cells, CD4^+^Foxp3^+^ T_Regs_, and CD11b^+^Ly6C^+^Ly6G^−^ MDSCs (256), demonstrating that the CAF secretome can modulate the recruitment and/or polarization of different immune subpopulations. In another model of aggressive lymphoma (Eµ-Myc), T cell zone resident stromal cells were shown to selectively retain CD4^+^ T cells in the TME to support tumor progression *via* CD40L signaling (237).

In FL, where tumor cells strongly depend on TME interactions, stromal-derived IL-6 supports the survival of T-follicular helper (T_FH_) and T-follicular regulatory (T_FR_) cells (257). T_FH_ cells, that sustain lymphoma through a number of mechanisms (258), were also shown to establish a feedback loop with stromal cells. IL-4, the predominant cytokine produced by T_FH_, was shown to trigger production of CXCL12 by FRC-like stromal cells, thus supporting FL tumor cell activation and survival (259). Additionally, FL-associated stroma (BM-MSCs) derived CCL2 and IL-8 were shown to promote the recruitment of monocytes/TAMs and neutrophils respectively, using *in vitro* assays (253, 260). Once in contact with FL-MSCs, monocytes/TAMs have been demonstrated to acquire an immunosuppressive phenotype with a reduced capacity to respond to pro-inflammatory stimuli such as LPS, while neutrophils supported the inflammatory stromal phenotype. Also in MM, malignant B cells have been shown to induce MSCs to secrete pro-tumor cytokines, as well thymic stromal lymphopoietin (TSLP) that activated T_H_2-type inflammation in the BM TME resulting in tumor progression (261) (**Figure 2B**).

Another important mechanism of how stromal cells contribute to an immunosuppressive TME is through the expression of immune checkpoint molecules. Lymphoma-derived ECs preferentially express TIM-3. Expression levels of TIM-3 in the B-cell lymphoma endothelium has been correlated with poor prognosis and TIM-3^+^ ECs suppressed the activation of CD4^+^ T cells inhibiting T_H_1 polarization *in vitro* and *in vivo* (273). In DLBCL, besides its expression on tumor and immune cells described earlier in this review, PD-L1 has also been associated to the non-malignant cellular compartment (“microenvironmental PD-L1”or mPD-L1) (262). Our own work has reported that PD-L1 upregulation on lymphoma-educated stromal cells can dampen TIL cytolytic killing activity against DLBCL tumor cells, highlighting that stromal cells expressing inhibitory ligands can directly modulate anti-tumor immune responses (274).

In addition to expression of immunomodulatory immune checkpoints, both LN and BM-derived stromal cells can adopt a number of other immunosuppressive mechanisms to modulate immune responses (**Figure 2B**). In MM, MSCs exhibit a distinctive gene expression profile compared to healthy MSCs, characterized by the expression of a number of immunoregulatory factors and cytokines including NOS2, IL-10, IL-6 and TGF-β, involved in the generation and activation of MDSCs (263, 264). In FL, MSCs also produce prostaglandin E2 (PGE2) that not only promotes neutrophil survival, but can have additional effect on other immune subpopulations (260). In physiological conditions, MSCs and LN-fibroblasts produce IDO to keep activated immune responses in check by inhibiting T cell proliferation, a mechanism also described in solid tumors (265). In FL, MSCs-derived IDO was also shown to repress not only T cell proliferation, but also the pro-lymphoma activity of stromal cells, highlighting the complex effects of cytokines in the TME (266). Clearly, our understanding of the immunomodulatory function of the different stromal cell populations that reside within lymphoma TMEs is still in its infancy. However, it is becoming clear that different non-hemopoietic cells within lymphoid TMEs can alter the recruitment, polarization and function of immune cells, suggesting that the tumor stroma may also influence immune responses elicited by current immunotherapy.

## Immune and Stroma Targeted Immunotherapy to Activate Anti-Lymphoma Activity

A number of studies have shown that the TME can interfere with clinical response to tumor-targeting therapy *via* different mechanisms. Reduced macrophage infiltration in the BM of ALL mice limits the response to anti-CD20 antibodies (alemtuzumab) (275), while adhesion to BM-derived stromal cells provides protection to CLL cells from rituximab-induced apoptosis (276). On the other side, tumor-targeting drugs can have indirect effects on the TME that may boost clinical responses. In CLL, there is evidence that ibrutinib treatment alleviates T cell exhaustion (277), while chemotherapy-induced cancer cell death promotes the exposure of tumor neoantigens and could re-invigorate anti-tumor immune responses (278). Understanding how the TME is shaped by currently available treatments can help to understand resistance mechanisms and to design combination therapies to boost clinical responses.

**Figure 3:**
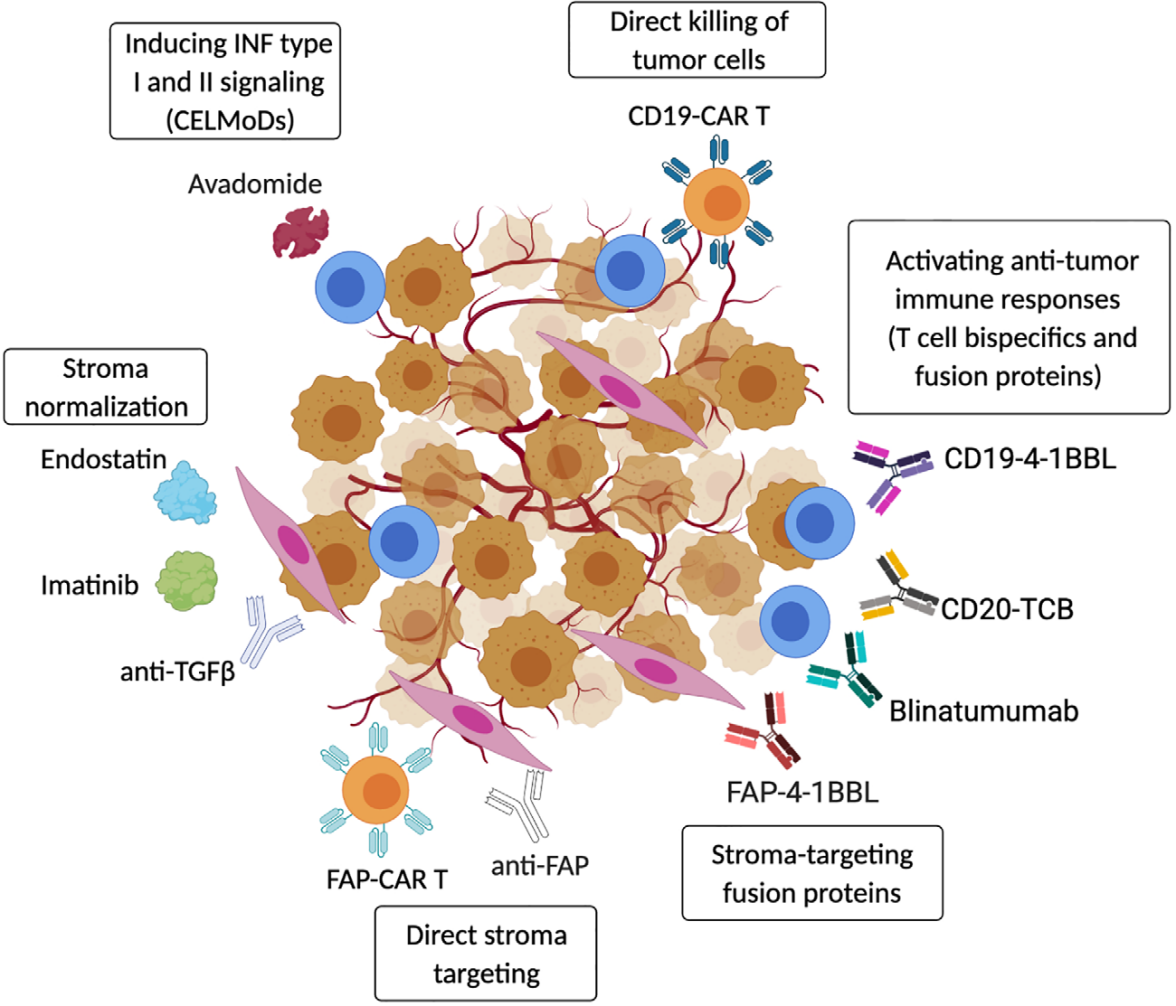
Targeting strategies to activate anti-tumor immunity in the TME. Therapeutic strategies include re-activation of autologous anti-tumor immune responses using CELMoDs (*e.g.* avadomide) (67) or T-cell bispecific antibodies and fusion proteins (CD20-TCB, CD19-4-1BBL, Blinatumumab) (279–282). Anti-tumor immunity can also be induced by transferring CAR-T/NK effector cells that directly target cancer cells (CD19-CAR-T) (283). In addition, cancer-associated stroma can be efficiently targeted to boost anti-tumor immunity, and fibroblasts-specific pharmacological approaches (anti-FAP antibodies or CAR-T) have been shown to induce tumor regression (284). Tumor stroma can also be ‘normalized’ by blocking or neutralizing cancer-secreted factors that promote stromal cell activation (Endostatin, Imatinib, anti-TGF*β*) (285–287). Moreover, the presence of cancer-stroma-specific proteins can be used to activate tissue resident anti-tumor T cells using stroma-specific/T cells co-stimulatory fusion proteins (FAP-4-1BBL) (282). (*Created with Biorender.com*).

The immuno-oncology era has introduced a vast array of drugs designed to engage and promote innate and adaptive immune responses within the TME. Thus, lymphoma therapy is changing dramatically with the introduction of several new therapeutic approaches, including the use of checkpoint inhibitors (3). However, as described here, only a subset of patients achieve long-lasting responses to anti-PD-1 monotherapy even if they harbor inflamed TMEs. This clinical experience, together with the lack of clinical activity of anti-PD-1 in NHL (108) and CLL (5), has highlighted the need to incorporate checkpoint blockade therapies into more powerful combinations to unleash the power of anti-tumor immune cells, with potential therapeutic partners including CELMoDs and immunomodulatory drugs, CAR T cells and bispecific antibodies (**Figure 3**).

As discussed earlier, there is substantial evidence demonstrating that type I and II IFN signaling is required within TMEs to prevent development of an immunosuppressive state (37, 49). Recent work by our group has demonstrated that the CELMoD avadomide can induce type I and II IFN signaling in the T cell compartment that sensitizes CLL to anti-PD-1 or anti-PD-L1 checkpoint blockers (67). Avadomide was shown to trigger a feedforward cascade of reinvigorated T cell responses, as well as IFN-inducible feedback inhibition through upregulation of PD-L1. Patient-derived xenograft tumor models revealed that inducing IFN-driven T cell responses with avadomide could convert non-inflamed CLL tumors into CD8^+^ T cell-inflamed TMEs that responded to anti-PD-L1/PD-1-based combination therapy. This pre-clinical study provides encouraging proof of concept that inducing inflammatory IFN type I and II signaling in patient T cells can successfully re-shape anti-tumor T cell responses and sensitize CLL to immunotherapy (67).

The re-activation of autologous anti-tumor immune responses has been also demonstrated in a number of different hematological malignancies by using “off the shelf” bispecific T cell engager (BiTE) antibodies. This dual binding of both neoplastic cells and tumor-infiltrating lymphocytes is providing an attractive therapeutic approach for B cell malignancies (279). Blinatumumab, a BiTE with dual specificity for CD3 and CD19, has shown activity in ALL and in different NHL, and has been approved by FDA in 2014 (280). However, due to its short half-life, Blinatumumab requires continuous infusion for weeks, causing patients discomfort. Moreover, the occurrence of different resistance mechanisms described for ALL patients (effector T cell exhaustion/dysfunction and expansion of T_Regs_), prompted the optimization and testing of alternative BiTE drugs in recent years [reviewed in (288)]. Among them, the T cell bispecific (TCB) antibody drug CD20-TCB (RG6026), with its 2:1 CD20-CD3 binding format, has shown superior potency in T cell activation and increased half-life in pre-clinical settings (281). CD20-TCB can be efficiently combined with the bispecific antibody fusion protein CD19-4-1BBL, that provides co-stimulatory signals to tumor-engaged immune cells (T cells or NK cells) and this combination immunotherapy has been shown to promote intratumoral TILs accumulation and increased anti-tumor efficacy in preclinical models of NHL (282). These pre-clinical results suggest that combination immunotherapy may achieve better clinical responses and overcome immune suppression in the TME. Currently, BiTEs and CD20 TCBs, including RG6026, are being evaluated in B cell malignancy patients with activity detected.

CD19-directed CAR-T cells have shown remarkable efficiency in the treatment of ALL and aggressive NHL (283, 289, 290) and, along the same line, new CD19-CAR-NK cells are showing effective anti-tumor activity with minor side effects (291). Indeed, CAR-T cell therapy has been the first FDA-approved cellular therapy in lymphoma [covered in a recent review (4)]. Despite this clinical success, emerging data is showing that durable responses induced by CAR Ts are seen in only a subset of patients and their anti-tumor activity depends on the persistence of CARs (181). While peripheral blood analysis of treated patients has shown that CAR-T concentrations peak at 14 days post-infusion (292), recent data has demonstrated that only a minimal amount of CAR-T cells infiltrate TME tissue at 5 days post-infusion, and that virtually no CAR-Ts were present in the TME 10 days following infusion (104). These results support the concept that the stroma-rich TME may contain ill-defined immunosuppressive mechanisms that interfere with the effective trafficking and optimal activation of anti-tumor immune cells using current immunotherapies.

Combining immunotherapy with drugs that target stromal cells is an attractive, next-generation treatment strategy. Different approaches can be adopted to target stromal cells in cancer including the direct targeting of fibroblasts/ECs or their associated proteins, activated signaling pathways or secreted factors. The majority of studies to date targeting tumor stroma have used solid cancer models, establishing a strong rationale for developing therapies for hematological malignancies. A strong case in point is fibroblast activating protein (FAP), a cell-surface serine protease which is expressed at high levels on tumor stroma and has been considered a suitable therapeutic candidate on CAFs for some time. Several FAP-specific pharmacological approaches (vaccines, CAR-T cells) designed to selectively target CAFs, were shown to induce tumor regression in different murine models (284). However, depletion of FAP^+^ fibroblasts has been shown to cause profound systemic immune-mediated toxicity, indicating their importance not only in cancer but also in physiological tissue functions (293). This example suggests that caution should be taken in considering direct elimination of stromal cells forming the TME given their critical functions in tissue architecture and immune homeostasis. Therefore, therapies designed to ‘normalize’ or ‘re-educate’ aberrant stromal cells by targeting or overcoming their pro-tumor, immune evasive pathways could be more clinically relevant (**Figure 3**).

To this direction, stromal cell normalization can be achieved by directly blocking or neutralizing cancer-secreted factors that promote stromal cell activation. To date, in lymphoma this approach has been exploited through the use of endostatin, an endogenous inhibitor of angiogenesis which inhibits matrix metalloproteinases (MMP) activity and blocks VEGF binding to VEGFR-2. Administration of endostatin in lymphoma-bearing mice was shown to delay tumor growth (285). Imatinib, a PDGFR*β* inhibitor, has also been shown to disrupt lymphoma angiogenesis by targeting vascular pericytes (286). Moreover, as PDGFR ligation also has a role in promoting fibroblast activation, the use of specific inhibitors can also affect fibroblast differentiation and function in the TME. As described above, TGF*β*, TNFα and LT are all master regulators of immunosuppressive fibroblast function in hematological malignancies, and the use of neutralizing antibodies against these molecules could provide an interesting approach for stroma normalization, as already demonstrated in solid tumors (294). Combined blockade of TGF*β* and anti-PD-1 in MM has been shown to promote anti-tumor T cell activation and proliferation, indicating that targeting the different immunosuppressive pathways occurring in the TME can reactivate endogenous anti-tumor immune responses and favor tumor clearance (287).

In conclusion, understanding the functional role of stroma and its specific features will help to design new combination immunotherapies to improve clinical responses. An example of effective immune-stroma dual targeting therapy comes from recent work from Claus and colleagues in solid tumors (282), where the combination of tumor antigen-TCB (CEA-TCB) with a stroma-specific/T cell co-stimulatory fusion protein (FAP-4-1BBL) promoted tumor remission and accumulation of activated CD8^+^ in the TME (**Figure 3**).

## Conclusions

Immunotherapy has revolutionized cancer treatment, achieving significant responses in patients with B cell malignancies, although sensitivity and resistance remain major challenges. Despite the extreme inflamed *vs* non-inflamed classification of immune TMEs, most B cell malignancies and their subtypes show a high degree of heterogeneity that likely influences responses to immunotherapy. For this reason, it is crucial for further research to fully unravel TME complexity including the major tumor intrinsic and extrinsic drivers of immune composition and spatial organization of immune and stromal cell subsets. Defining how malignant B cells alter both immune and stromal cells, as well as how these reprogrammed cells contribute to the creation of a pro-tumor and immunosuppressive TME will be essential to design next-generation immunotherapies and combination treatment strategies to overcome TME-driven immune suppression and optimize therapy for patients.

## Author Contributions

All authors contributed to the article and approved the submitted version.

## Conflict of Interest

AGR has received research funding to Institution from Bristol-Myers Squibb Roche Glycart AG and AstraZeneca.

The remaining authors declare that the research was conducted in the absence of any commercial or financial relationships that could be construed as a potential conflict of interest.

## Abbreviations

CLL, chronic lymphocytic leukemia; SLL, small lymphocytic lymphoma; DLBCL, diffuse large B-cell lymphoma; cHL, classical non-Hodgkin lymphoma; NHL, non-Hodgkin lymphoma; MM, multiple myeloma; PMBL, primary mediastinal B-cell lymphoma; FL, follicular lymphoma; MCL, mantle-cell lymphoma; ALL, acute lymphoblastic leukemia; CRC, colorectal carcinomas; EBV, Epstein–Barr virus; GCB, non-germinal center B-cell subtype; R/R relapsed/refractory; ORR, overall response rate; TME, tumor microenvironment; LN(s), lymph nodes; BM, bone marrow; TILs, tumor-infiltrating lymphocytes; TAMs, tumor-associated macrophages; APCs, antigen-presenting cells; DCs, dendritic cells; NK(s), natural killer cells; FDCs, follicular dendritic cells; MDSCs, myeloid-derived suppressor cells; ECs, endothelial cells; TECs, tumor-associated endothelial cells; LECs, lymphatic endothelial cells; BECs, blood endothelial cells; HEVs, high endothelial venules; FRCs, fibroblastic reticular cells; CAFs, cancer-associated fibroblasts; MSCs, mesenchymal stromal cells; PD-1, programmed cell death protein 1; IFN, interferon; HLA, human leukocyte antigen; IL-, Interleukin-; MHC, major histocompatibility complex; TRAIL, TNF-related apoptosis-inducing ligand; CELMoD, cereblon E3 ligase modulator; IRF4, IFN regulatory factor 4; CTLA-4, cytotoxic T-lymphocyte-associated protein 4; CAR T cells, chimeric antigen receptor T cells; JAK2, Janus kinase 2; NF-kB, nuclear factor kappa-light-chain-enhancer of activated B cells; STAT3, signal transducer and activator of transcription 3; MAPK, mitogen-activated protein kinase; PTEN, phosphatase and tensin homolog; EZH2, enhancer of zeste homolog 2; TP53, tumor protein P53; BCL2, B-cell lymphoma 2; TGF-*β*, transforming growth factor beta; EVs, extracellular vesicles; ARG1, arginase 1; IDO, indoleamine-pyrrole 2,3-dioxygenase; LAG-3, lymphocyte activation gene-3; TIM-3, T-cell immunoglobulin and mucin-domain containing-3; FOXP3, forkhead box p3; NO, nitric oxide; ICAM-1, intercellular adhesion molecule 1; VCAM-1, vascular cell adhesion protein 1; CXCL, C-X-C motif chemokine ligand; CXCR, C-X-C motif chemokine receptor; CCR, (C-C motif) chemokine receptor; SDF-1, stromal cell-derived factor 1; MCP-1, monocyte chemoattractant protein-1; Chi3L1, chitinase 3 like 1; ECM, extracellular matrix; PGE2, prostaglandin E2; VEGF, vascular endothelial growth factor; *α*SMA, alpha smooth muscle actin; (R-)CHOP, (rituximab-), cyclophosphamide, doxorubicin hydrochloride (hydroxydaunorubicin), vincristine sulfate (Oncovin), and prednisone; PDGF, platelet-derived growth factor; BAFF, B-cell activating factor; LT, lymphotoxin; TSLP, thymic stromal lymphopoietin; NOS2, nitric oxide synthase 2; FAP, fibroblast activating protein.

## Glossary

**CLL**: **chronic lymphocytic leukemia**
**SLL**: **small lymphocytic lymphoma**
**DLBCL**: **diffuse large B-cell lymphoma**
**cHL**: **classical non-Hodgkin lymphoma**
**NHL**: **non-Hodgkin lymphoma**
**MM**: **multiple myeloma**
**PMBL**: **primary mediastinal B-cell lymphoma**
**FL**: **follicular lymphoma**
MCL: mantle-cell lymphoma
ALL: acute lymphoblastic leukemia
CRC: colorectal carcinomas
EBV: Epstein–Barr virus
GCB: non-germinal center B-cell subtype
R/R: relapsed/refractory
ORR: overall response rate
TME: tumor microenvironment
LN(s): lymph nodes
BM: bone marrow
TILs: tumor-infiltrating lymphocytes
TAMs: tumor-associated macrophages
APCs: antigen-presenting cells
DCs: dendritic cells
NK(s): natural killer cells
FDCs: follicular dendritic cells
MDSCs: myeloid-derived suppressor cells
ECs: endothelial cells
TECs: tumor-associated endothelial cells
LECs: lymphatic endothelial cells
BECs: blood endothelial cells
HEVs: high endothelial venules
FRCs: fibroblastic reticular cells
CAFs: cancer-associated fibroblasts
MSCs: mesenchymal stromal cells
PD-1: programmed cell death protein 1
IFN: interferon
HLA: human leukocyte antigen
IL-: Interleukin-
MHC: major histocompatibility complex
TRAIL: TNF-related apoptosis-inducing ligand
CELMoD: cereblon E3 ligase modulator
IRF4: IFN regulatory factor 4
CTLA-4: cytotoxic T-lymphocyte-associated protein 4
CAR T cells: chimeric antigen receptor T cells
JAK2: Janus kinase 2
NF-kB: nuclear factor kappa-light-chain-enhancer of activated B cells
STAT3: signal transducer and activator of transcription 3
MAPK: mitogen-activated protein kinase
PTEN: phosphatase and tensin homolog
EZH2: enhancer of zeste homolog 2
TP53: tumor protein P53
BCL2: B-cell lymphoma 2
TGF-*β*: transforming growth factor beta
EVs: extracellular vesicles
ARG1: arginase 1
IDO: indoleamine-pyrrole 2,3-dioxygenase
LAG-3: lymphocyte activation gene-3
TIM-3: T-cell immunoglobulin and mucin-domain containing-3
FOXP3: forkhead box p3
NO: nitric oxide
ICAM-1: intercellular adhesion molecule 1
VCAM-1: vascular cell adhesion protein 1
CXCL: C-X-C motif chemokine ligand
CXCR: C-X-C motif chemokine receptor
CCR: (C-C motif) chemokine receptor
SDF-1: stromal cell-derived factor 1
MCP-1: monocyte chemoattractant protein-1
Chi3L1: chitinase 3 like 1
ECM: extracellular matrix
PGE2: prostaglandin E2
VEGF: vascular endothelial growth factor
*α*SMA: alpha smooth muscle actin
R-)CHOP: (rituximab-) cyclophosphamide doxorubicin hydrochloride (hydroxydaunorubicin) vincristine sulfate (Oncovin) and prednisone
PDGF: platelet-derived growth factor
BAFF: B-cell activating factor
LT: lymphotoxin
TSLP: thymic stromal lymphopoietin
NOS2: nitric oxide synthase 2
FAP: fibroblast activating protein
